# Neuroprotective Effect of Natural Compounds in Paclitaxel-Induced Chronic Inflammatory Pain

**DOI:** 10.3390/molecules27154926

**Published:** 2022-08-02

**Authors:** Muhammad Faheem, Arif-ullah Khan, Muhammad Waqas Saleem, Fawad Ali Shah, Fawad Ali, Abdul Waheed Khan, Shupeng Li

**Affiliations:** 1 Riphah Institute of Pharmaceutical Sciences, Riphah International University, Islamabad 45000, Pakistan; fawad.shah@riphah.edu.pk; 2 Rural Health Centre, Head RajkanYazman, Bahawalpur 63236, Pakistan; waqassaleem2255@gmail.com; 3 Department of Pharmacy, Kohat University of Science and Technology, Kohat 26000, Pakistan; fawad.alee@gmail.com; 4 Department of Molecular Science and Technology, Ajou University, Suwon 16499, Korea; waheedmarwat31@gmail.com; 5 State Key Laboratory of Oncogenomics, School of Chemical Biology and Biotechnology, Shenzhen Graduate School, Peking University, Shenzhen 518000, China

**Keywords:** berbamine, bergapten, carveol, paclitaxel, ELISA, Western blot

## Abstract

The current study explored the effects of natural compounds, berbamine, bergapten, and carveol on paclitaxel-associated neuroinflammatory pain. Berbamine, an alkaloid obtained from *Berberis* *amurensisRuprhas* been previously researched for anticancer and anti-inflammatory potential. Bergapten is 5-methoxsalenpsoralen previously investigated in cancer, vitiligo, and psoriasis. Carveol obtained from caraway is a component of essential oil. The neuropathic pain model was induced by administering 2 mg/kg of paclitaxel (PTX) every other day for a week. After the final PTX injection, a behavioral analysis was conducted, and subsequently, tissue was collected for molecular analysis. Berbamine, bergapten, and carveol treatment attenuated thermal hypersensitivity, improved latency of falling, normalized the changes in body weight, and increased the threshold for pain sensation. The drugs increased the protective glutathione (GSH) and glutathione S-transferase (GST) levels in the sciatic nerve and spinal cord while lowering inducible nitric oxide synthase (iNOS) and lipid peroxidase (LPO). Hematoxylin and eosin (H and E) and immunohistochemistry (IHC) examinations confirmed that the medication reversed the abnormal alterations. The aforementioned natural substances inhibited cyclooxygenase-2 (COX-2), tumor necrosis factor-alpha (TNF-α), and nuclear factor kappa B (NF-κb) overexpression, as evidenced by enzyme-linked immunosorbant assay (ELISA) and Western blot and hence provide neuroprotection in chronic constriction damage.

## 1. Introduction

Paclitaxel is well-known for its anti-tumor properties [1]. Chemotherapy is one of the most effective techniques for fighting cancer and enhancing the quality of life, resulting in fewer deaths throughout the globe. With appropriate use of chemotherapy, researchers predict a 35 percent increase in cancer survivors by 2022 [2]. One of the most prevalent dose-limiting side effects associated with the use of anticancer medicines is peripheral neuropathy [3].

Taxanes are a family of significant chemotherapeutic drugs that have been therapeutically used for decades to treat a variety of malignancies. Paclitaxel is a taxane-class medication that has been authorized by the FDA to treat lung cancer, breast cancer, prostate cancer, and ovarian cancer [4]. Its use has been linked to the development of dose-dependent neurotoxicity. The termination of treatment is due to the development of cold allodynia, mechanical hypersensitivity, and burning, shooting, and tingling sensations [5].

According to the research, the mechanism of PTX-induced neurotoxicity is thought to be initiated by the overexpression of inflammatory mediators in the spinal cord and the sciatic nerve. This overexpression leads to the disruption of transport in ion channels and improper intracellular signaling, which ultimately results in paclitaxel-induced peripheral neuropathy (PIPN) [6]. Recently published data show that because PTX can easily pass through the blood–brain barrier, it predominantly accumulates in the dorsal root ganglia (DRG). This, along with an alteration in the mitochondrial morphology and inflammation, leads to the development of PIPN, which causes cold allodynia as well as mechanical and thermal pain sensation [7,8].

The present study aims to look at the therapeutic potential of natural substances including berbamine, bargepten, and carveol in the treatment of paclitaxel-induced neuropathic pain. Herbal ingredients have been studied extensively for their ability to reduce paclitaxel-induced pain. Natural treatments such as curcumin [9], resveratrol [10], gallic acid [11], puerarin [12], and naringin [13] have been shown to reduce paclitaxel-induced discomfort. Chemically, berbamine (BBM) is a bisbenzylisoquinoline alkaloid obtained from the Chinese medicinal plant *Berberis amurensis Rupr*. It has anti-cancer, anti-inflammatory, and multidrug resistance properties, as well as a synergistic effect when combined with other medications [14].

Psoralen is a group of natural substances derived from the Ammi majus plant that are collectively known as Furocoumarin. Furocoumarin is a methoxsalen-based Furocoumarin. Bergapten (BRG) is a 5-methoxsalenpsoralen compound that has previously been studied in cancer, vitiligo, and psoriasis [15]. Carveol (CAR) is derived from the Caraway plant. It is a vital component of essential oil and is grown all over the globe. Caraway also contains pinene, thujene, phellandrene, camphene, limonene, and carvone as components [16].

To investigate the potential in PTX-induced PIPN, the above-mentioned three natural moieties (BBM, BRG, and CAR) listed were chosen. The available literature supports their potential for anticancer [14,15] and neuroprotective properties [17].

Hence, these compounds were examined in the current study for their therapeutic potential in PTX-induced peripheral and chronic inflammatory pain. The results reveal that treatment with natural compounds (BBM, BRG, and CAR) attenuated PTX-induced chronic inflammatory pain by downregulating NF-κB. The downregulation of NF-κB, a transcription factors which further attenuates inflammatory cytokines (COX-2 and TNF-α), is the proposed mechanistic pathway of the aforementioned natural compounds to hasten PTX-induced neuropathic pain. In addition, the treatment improved the antioxidant enzymes (GSH and GST) and diminished LPO and iNOS, which are the reasons of preventing oxidative stress and free radical generation to stop the PTX-induced progression of neurodegeneration and neuroinflammation.

## 2. Material and Methods

### 2.1. Chemicals

Proteinase K, PBS tablets, hydrogen peroxide (H_2_O_2_), formaldehyde, GSH, DTNB, CDNB, Mouse monoclonal anti-p-NF-κB, TNF-α, COX-2, Avidin-biotin complex kit, DAB, trichloroacetic acid (TCA), horseradish peroxidase-conjugated secondary antibodies, mounting media, COX-2, p-NF-κB, TNF-α ELISA and protein assay kit, skim milk and Bolt Mini Gels, X-ray film were used. The inducer paclitaxel was procured from the oncology pharmacy of Shifa International Hospital Islamabad, Islamabad, Pakistan. All the rest of the three compounds were procured from Sigma-Aldrich (Saint Louis, MO, USA).

### 2.2. Animals

Adult Sprague Dawley rats weighing 240–250 g (aged 10–12 weeks) were procured from Riphah International University’s animal house (Islamabad, Pakistan) kept under in a controlled environment temperature (25–30 °C) and humidity. The experimental protocols for handling and dosing of animal were as per protocols set bythe Riphah Institute of Pharmaceutical Sciences (Ref. No. REC/RIPS/2019/28) (Islamabad, Pakistan).

The animals were randomly divided into five groups, each group with six rats as follows:

**Group** **I:** Control group, treated with saline 10 mL/kg

**Group** **II:** Disease group, treated with PTX 2 mg/kg IP on days 1, 3, 5, and 7 (induction phase)

**Group** **III:** Treated group, administered with PTX 2 mg/kg IP on days1, 3, 5, and 7 (induction phase) and then treated with compound (berbamine) for two weeks (7 to 21 days)

**Group** **IV:** Treated group, administered with PTX 2 mg/kg IP on days 1, 3, 5, and 7 (induction phase) and then treated with compound (bergapten) for two weeks (7 to 21 days)

**Group** **V:** Treated group, administered with PTX 2 mg/kg IP on days 1, 3, 5, and 7 (induction phase) and then treated with compound (carveol) for two weeks (7 to 21 days)

### 2.3. Paclitaxel-Induced Neuropathic Pain

Adult male Sprague Dawley rats were injected with paclitaxel to produce paclitaxel-induced peripheral neuropathic pain. The available pharmaceutical grade of paclitaxel was 6 mg/mL, which was further diluted (1:1 cremophor/ethanol) to 1 mg/mL and injected intraperitoneally (i.p) at a dose of 2 mg/kg every other day (1, 3, 5, 7) for a total of four injections and a final total dose of 8 mg/kg. On days 7, 14, and 21 (days after PTX’s last injection), behavioral tests including temperature sensation, latency of falling, body weight, and mechanical pain threshold were performed. After behavioral tests on day 21, the sciatic nerve (SN) and spinal cord (SC) were removed for molecular investigation [18].

### 2.4. Thermal Pain Sensation

Acclimatized animal’s paw sensitivity to heat was determined on a hot plate (54 ± 1 °C) with a cut-off time of time of 60 s. Thermal pain sensation was determined on days 7, 14, and 21, 30 min after treatment with the compound [19,20].

#### 2.4.1. Latency of Falling

The latency of falling was recorded through the rotarod apparatus. Animals were first trained for 3 consecutive days to be able to remain on the rod for 60 s with 5 min cut-off time before starting the experiment. The animals were at a fixed rate (5–20 rpm). After recording the baseline reading (day 0), the latency of falling was recorded. Latency of falling was also determined on days 7, 14, and 21after treatment with compound [21].

#### 2.4.2. Body Weight Analysis

The animals were first weighed at baseline before starting the experiments. The weight of each animal was recorded after therapy on days 7, 14, and 21, and the results were interpreted [22].

#### 2.4.3. Pain Threshold (Mechanical Hypersensitivity)

A Conventional von-Frey filament apparatus was used to analyze the pressure-induced-pain threshold [23]. After its perpendicularly application to the subplanter region, the response of the animal was noticed on days 7, 14, and 21after treatment.

### 2.5. Oxidative Stress Markers

The samples (SN and SC) after homogenization in a phosphate buffer containing phenylmethylsulfonyl fluoride as a protease inhibitor were centrifuged at 4000× *g* for 10 min at 4 °C, and the supernatant was collected and processed for determination of GSH [24] and GST [25], lipid peroxidation [26], and nitric oxide (NO) [27].

### 2.6. Hematoxylin and Eosin Staining

De-paraffinization was performed through xylene (100%), ethanol (95 and 70%), and distilled water. There was washing with PBS and treatment with hematoxylin for 10 min. Each slide was dipped in 1% HCl, immersed in eosin for 5–10 min, washed, dried, and fixed in xylene. The images were acquired with the assistance of an Olympus (Model: CX31, Tokyo, Japan) microscope, and an automated self-quantification process was carried out with the assistance of ImageJ software (USA, version 1.46) [28].

### 2.7. Immuno-Histopathological Evaluation

Paraffinized slides were washed with xylene, ethanol, distilled water, and then in PBS. Proteinase K was applied for antigen retrieval. Endogenous peroxidases were blocked by hydrogen peroxide. Normal goat serum (5%), primary antibody secondary antibody, and ABC were then applied. Finally, the slides were exposed to a 0.1% DAB (diaminobenzidine peroxidase) solution and dehydrated by dipping in xylene/ethanol and dried in open air. The images were obtained through an Olympus (Model: CX31, Tokyo, Japan) microscope and an automated self-quantification method was applied utilizing ImageJ software (USA, version 1.46) [29].

### 2.8. ELISA

TNF-α, COX-2, and NF-κB were determined by using ELISA kits as explained by [30]. In the tissue, the designated antibodies’ expression was determined by using an ELISA microplate reader.

### 2.9. Western Blot Assay

Equal amounts of protein (15–30 μg) underwent electrophoresis using 4–12% bolt Mini Gels 5% (*w*/*v*).Following the wet transfer, the PVDF membrane was blocked with skim milk to reduce nonspecific binding and incubated with primary antibodies at 4 °C for 12 to 16 h. After a reaction with a horse radish peroxidase (HRP)-conjugated secondary antibody, proteins were detected using enhanced chemiluminescence detection. The X-ray films were scanned, and the optical densities of the bands were analyzed through densitometry using the computer-based Sigma Gel program, version 1.0 (USA) [31].

### 2.10. Statistical Analysis

Data are presented as the mean ± SEM. H and E staining behavioral data and oxidative stress data were analyzed using one-way ANOVA, followed by post hoc Tukey’s test using GraphPad Prism version 6.0 (San Diego, CA, USA). The *p*-value was calculated through GraphPad Instat software version 3.1 (San Diego, CA, USA). ImageJ software was used to analyze the morphological data. One-way ANOVA followed by post hoc Tukey’s test was performed for ELISA and Western blot. Symbols ^#^ or * represent significant difference values *p* < 0.05, ^##^ or ** represent *p* < 0.01, and ^###^ or *** represent *p* < 0.001 values.

## 3. Results

### 3.1. Effect on Thermal Pain Sensation

**Figure 1:**
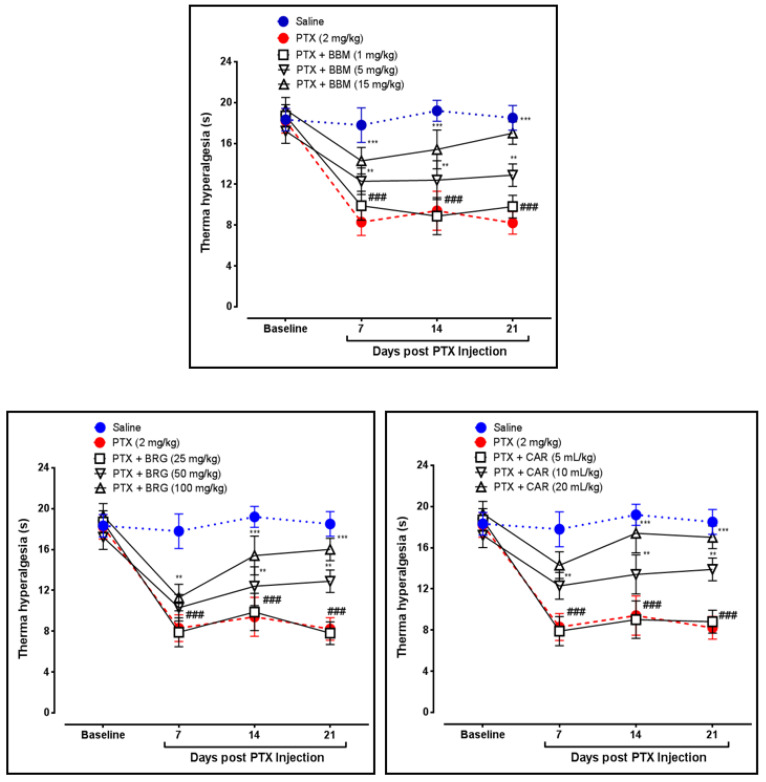
Line graph representing the effect of berbamine (1, 5, and 15 mg/kg), bargepten (25, 50, and 100 mg/kg), and carveol (5, 10, and 20 mL/kg) on thermal hyperalgesia on 7th, 14th, and 21st days. The data are expressed as the mean ± SEM, *n* = 6. One-way ANOVA with posthoc Tukey’s test. ** *p* < 0.01 and *** *p* < 0.001 indicate a significant difference vs. PTX, and ^###^ *p* < 0.001 indicates a significant difference vs. saline.

Berbamine elevated heat latency (HL) at 5 and 15 mg/kg on days 14 and 21, respectively, as compared to PTX. Bergapten at 50 and 100 mg/kg enhanced HL on days 14 and 21, respectively, vs. PTX. Carveol at 10 and 20 mL/kg increased HL on days 14 and 21, respectively, vs. PTX. PTX at 2 mg/kg decreased HL on days 7, 14, and 21 vs. control, presented in Figure 1.

### 3.2. Effect on the Latency of Falling

**Figure 2:**
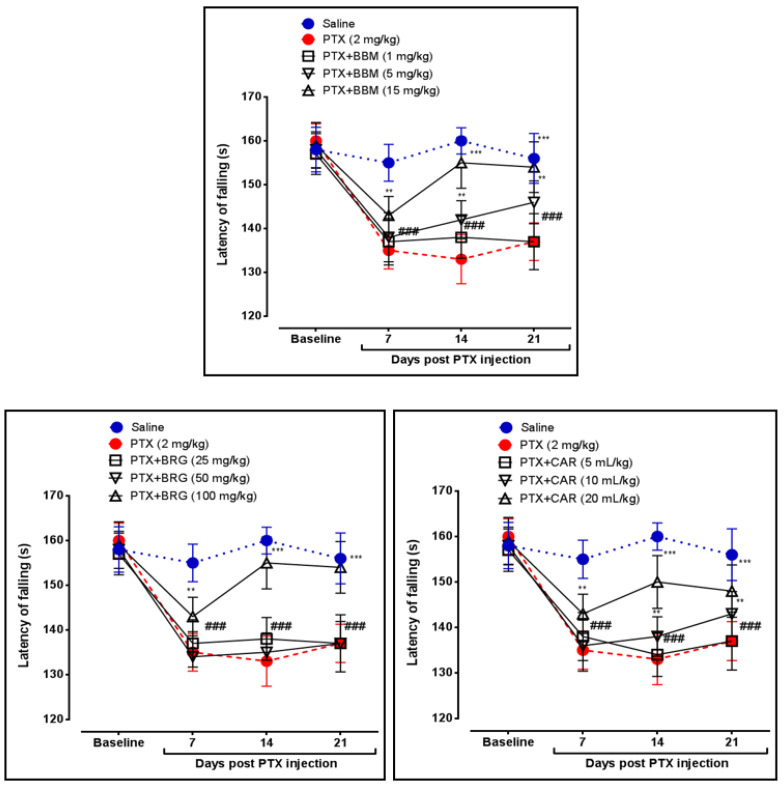
Line graph representing the effect of berbamine (1, 5, and 15 mg/kg), bargepten (25, 50, and 100 mg/kg), and carveol (5, 10, and 20 mL/kg) on latency of falling on 7th, 14th, and 21st days. The data are expressed as the mean ± SEM, *n* = 6. One-way ANOVA with posthoc Tukey’s test. ** *p* < 0.01 and *** *p* < 0.001 indicate a significant difference vs. PTX, and ^###^ *p* < 0.001 indicates a significant difference vs. saline.

Latency of falling (LF) or motor coordination was determined by the rotarod apparatus. Berbamine at 5 and 15 mg/kg augmented LF on days 14 and 21, respectively, vs. PTX. Bergapten at 100 mg/kg enhanced LF on days 14 and 21 vs. PTX. Carveol at 10 and 20 mL/kg raised LF on days 14 and 21, respectively, vs. PTX. PTX at 2 mg/kg decreased LF on days 7, 14, and 21 vs. that in the control group, presented in Figure 2.

### 3.3. Effect on Changes in Body Weight

**Figure 3:**
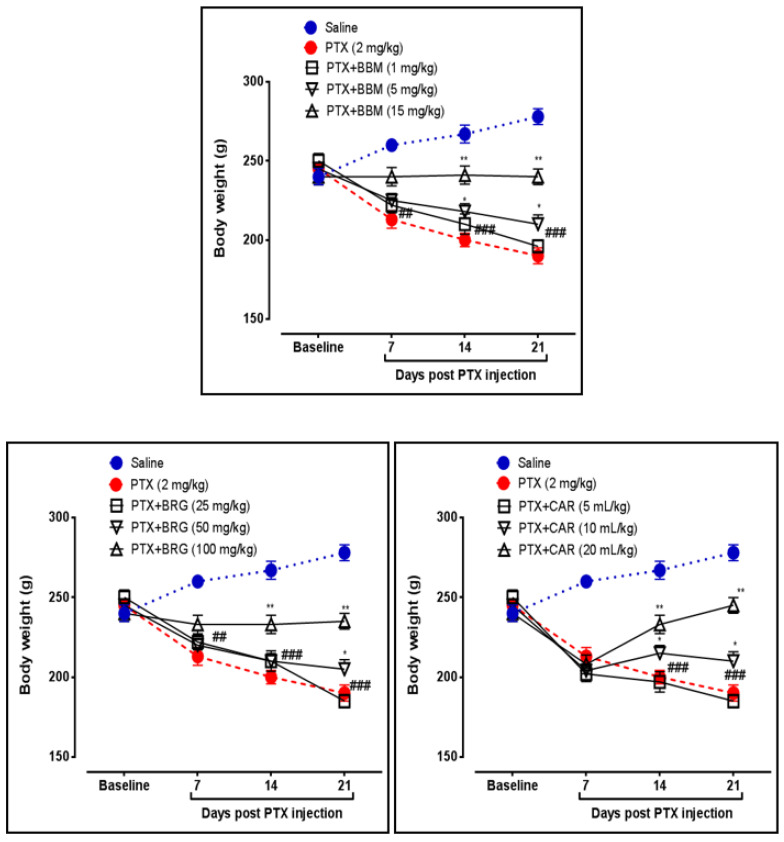
Line graph representing the effects of berbamine (1, 5, and 15 mg/kg), bargepten (25, 50, and 100 mg/kg), and carveol (5, 10, and 20 mL/kg) on body weight on 7th, 14th, and 21st days. The data are expressed as the mean ± SEM, *n* = 6. One-way ANOVA with post hoc Tukey’s test. * *p* < 0.05 and ** *p* < 0.01 indicate a significant difference vs. PTX, and ^###^ *p* < 0.001 indicates a significant difference vs. saline.

Changes in body weight were also determined by utilizing weighing balance. Berbamine increased body weight at 5 and 15 mg/kg on days 14 and 21, respectively, vs. PTX. Bergapten increased body weight at 50 mg/kg on day 21 vs. PTX. Bergapten increased body weight at 100 mg/kg on days 14 and 21 vs. PTX. Carveol increased body weight at 10 and 20 mL/kg on days 14 and 21 vs. PTX. PTX at 2 mg/kg decreased the latency of falling on days 7, 14, and 21 vs. that in the control, presented in Figure 3.

### 3.4. Effect on vonFrey-Induced Pain

**Figure 4:**
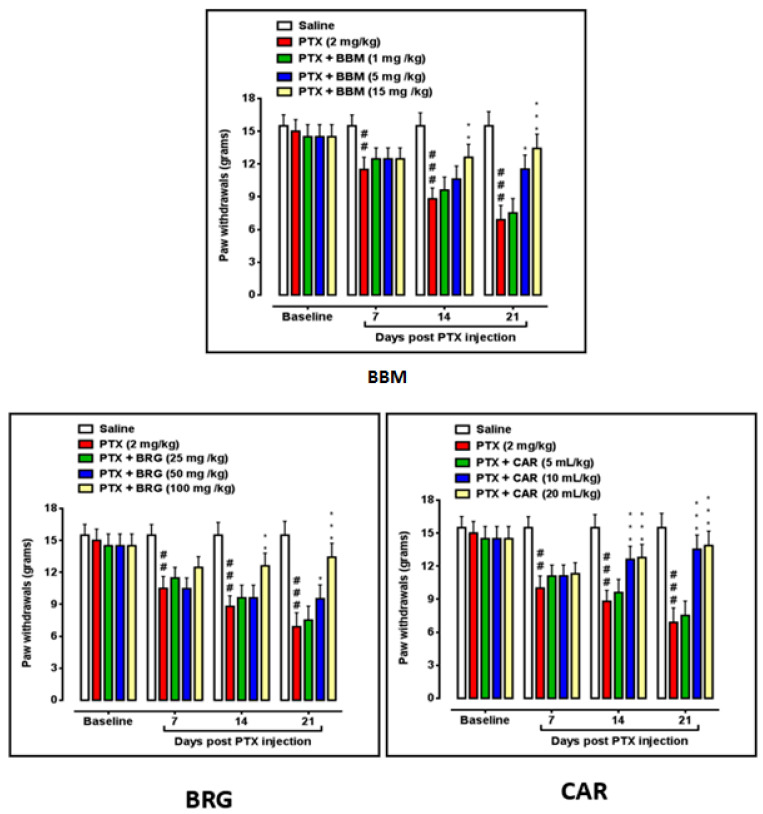
Bar graph representing the effects of berbamine (1, 5, and 15 mg/kg), bargepten (25, 50, and 100 mg/kg), and carveol (5, 10, and 20 mL/kg) on mechanical hypersensitivity on 7th, 14th, and 21st days. The data are expressed as the mean ± SEM, *n* = 6. One-way ANOVA with post hoc Tukey’s test. * *p* < 0.05, ** *p* < 0.01, and *** *p* < 0.001 indicate a significant difference vs. PTX, and ^##^ *p* < 0.01 and ^###^ *p* < 0.001 indicate a significant difference vs. saline.

Berbamine increased the paw withdrawal threshold (PWT) at 5 mg/kg on day 21 vs. PTX. At 15 mg/kg, berbamine improved PWT on day 14 vs. PTX. Berbamine at 15 mg/kg, augment PWT on day 21 vs. PTX. Bergapten at 50 mg/kg increased PWT on day 21 vs. PTX. Bergapten at 100 mg/kg elevated PWT on day 14 vs. PTX. Bergapten at 100 mg/kg, augmented PWT on day 21 vs. PTX. Carveol at 10 mL/kg raised paw PWT on days 14 and 21 vs. PTX. Carveol at 20 mL/kg increased PWT on days 14 and 21 vs. PTX. PTX at 2 mg/kg decreased PWT on days 7, 14, and 21 with *p* < 0.001 vs. that in the control, presented in Figure 4.

### 3.5. Effect on Oxidative Stress Enzyme

Berbamine, bergapten, and carveol were tested for their ability to inhibit oxidative-stress-causing enzymes. In the PTX-induced neuropathic pain group, PTX dramatically loweredthe levels of GSH and GST. In the SN and SC, berbamine dramatically boosted the protective markers GSH and GST. In both SN and SC, bergapten enhanced GSH and GST levels. Carveol reduced free radical production by increasing GSH and GST in the SN and SC, as seen in Table 1 and Table 2. Berbamine, bergapten, and carveol were studied for their effects on iNOS and LPO. Destructive oxidative agents such as iNOS and LPO were observed to be elevated in the PTX group. In the SN and SC, berbamine significantly reduced iNOS and LPO. In the SN and SC, bergapten dramatically reduced iNOS and LPO. Carveol decreased iNOS and LPO in the SN and downregulated iNOS and LPO in the SC, as seen in Table 1 and Table 2.

### 3.6. H and E Staining Examination

**Figure 5:**
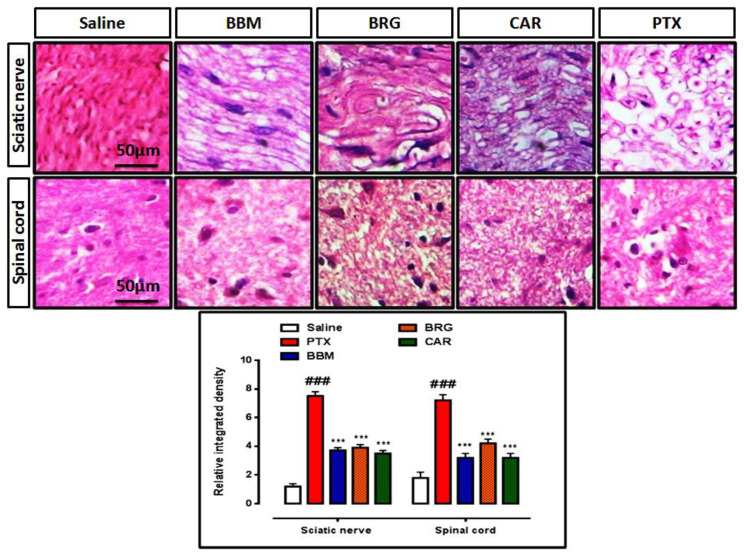
Representation of hematoxylin and eosin staining and the effects of berbamine, bargepten, and carveol on PTX-induced alteration in the sciatic nerve and spinal cord. The data are expressed as the mean ± SEM, *n* = 6. One-way ANOVA with posthoc Tukey’s test. *** *p* < 0.001 indicates a significant difference vs. PTX, and ^###^ *p* < 0.001 indicates a significant difference vs. saline. Morphological data were analyzed by ImageJ software. Bar 50 µm, magnification 40×.

H and E staining demonstrated an organized cellular architecture, no infiltration, and intact intracellular spaces with no evidence of edema in the saline group. In the PTX group, compared to the saline group, PTX-induced pathological abnormalities, damage of the SN and SC with different types of injuries in the form of enlarged intracellular spaces, infiltration, and edema of a disorderly pattern were seen. As demonstrated in Figure 5, treatment with berbamine, bergapten, and carveol dramatically restored the PTX-induced damage and pathological development in the SN and SC compared to those in the PTX group.

### 3.7. IHC Analysis

**Figure 6:**
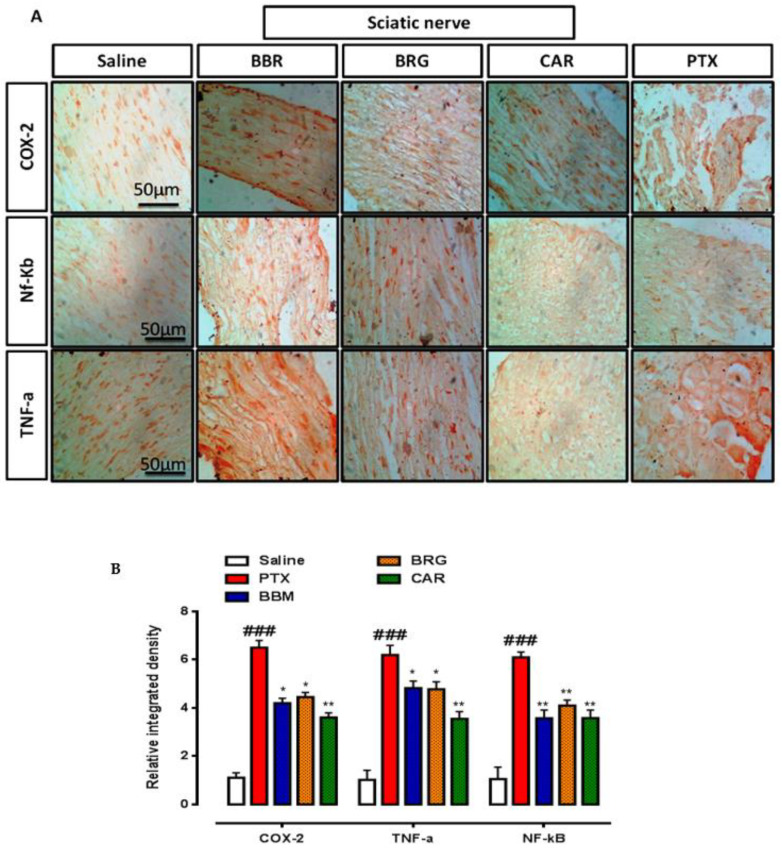
(**A**) Representation of immunohistochemistry results for COX-2, TNF-α, and p-NF-κbin the sciatic nerve of the rat. (**B**) Histograms showed comparatively higher expression of COX-2, TNF-α, and p-NF-κbin the PTX group. The data are expressed as the mean ± SEM, *n* = 6. One-way ANOVA with posthoc Tukey’s test. * *p* < 0.05 and ** *p* < 0.01 indicate a significant difference vs. PTX, and ^###^ *p* < 0.001 indicates a significant difference vs. saline. Morphological data were analyzed by ImageJ software. Bar 50 µm, magnification 40×.

**Figure 7:**
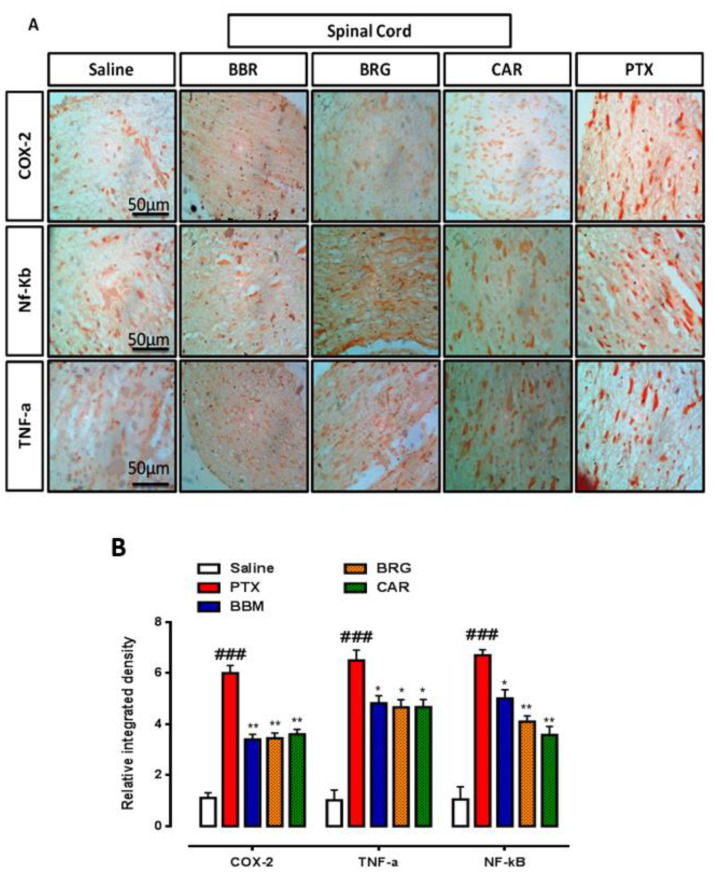
(**A**) Representation of immunohistochemistry results for COX-2, TNF-α, and p-NF-κbin the spinal cord of rat. Bar 50 µm, magnification 40x (*n* = 6/group). (**B**) Histograms showed comparatively higher expression of COX-2, TNF-α, and p-NF-κb in the PTX group. The data are expressed as the mean ± SEM, *n* = 6. One-way ANOVA with posthoc Tukey’s test. * *p* < 0.05 and ** *p* < 0.01 indicate a significant difference vs. PTX, and ^###^ *p* < 0.001 indicates a significant difference vs. saline. Morphological data were analyzed by ImageJ software. Bar 50 µm, magnification 40×.

The findings of IHC staining are presented in Figure 6 and Figure 7. COX-2, TNF-α, and p-NF-κb were notably seen raised in the PTX group compared to those in the saline group in the SN and SC. Berbamine attenuated COX-2, TNF-α, and p-NF-κb significantly in the SN. Bergapten vanished COX-2, TNF-α, and p-NF-κb significantly in the SN. Carveol suppressed COX-2, TNF-α, and NF-κb significantly in the SN, presented in Figure 6A,B. Berbamine attenuated COX-2, TNF-α, and p-NF-κb significantly in the SC. Bergapten downregulated COX-2, TNF-α, and p-NF-κb significantly in the SC. Carveol reduced COX-2, TNF-α, and p-NF-κb significantly in the SC, presented in Figure 7A,B.

### 3.8. Effects on Inflammatory Marker (ELISA)

**Figure 8:**
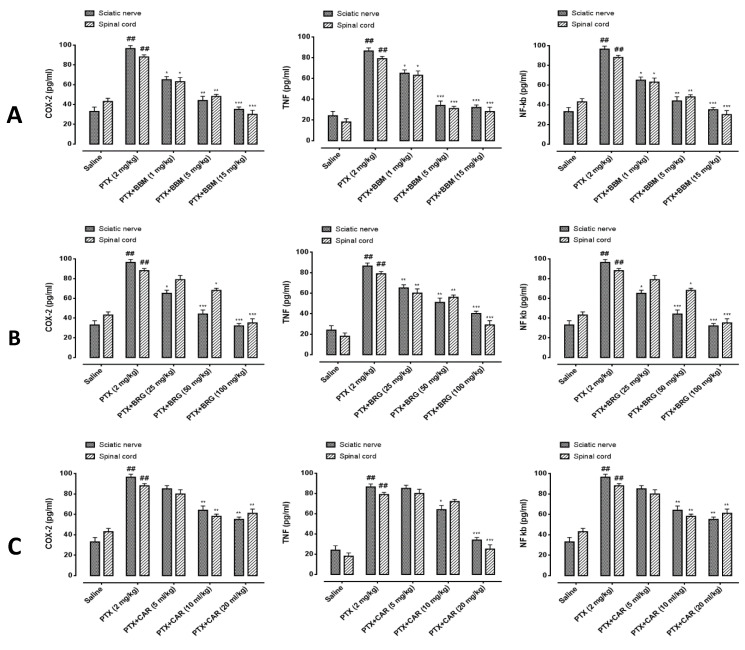
Representation of the effect of berbamine (**A**), bergapten (**B**), and carveol (**C**) on the expression of COX-2, TNF-α, and NF-κb in the sciatic nerve and spinal cord, quantified by using enzyme-linked immunosorbent assays. The data are expressed as the mean ± SEM, *n* = 6. One-way ANOVA with posthoc Tukey’s test. * *p* < 0.05, ** *p* < 0.01, and *** *p* < 0.001 indicate a significant difference vs. PTX, and ^##^ *p* < 0.01 indicates a significant difference vs. saline.

As shown in Figure 8, we studied the effects of berbamine, bergapten, and carveol on the expression of COX-2, TNF-α, and p-NF-κb. All three mediators were found raised in the PTX group vs. saline in the SN and SC.Berbamine at 1 mg/kg minimized expression of COX-2 in the SN and SC. At 5 mg/kg, it suppressed COX-2 expression in the SN and SC, at 15 mg/kg, it reduced COX-2 expression in the SN and SC. Bergapten at 25 mg/kg decreased COX-2 in the SN. At 50 mg/kg it downregulated COX-2 in the SN, and at 50 mg/kg it reduced COX-2 in the SC. Bergapten at 100 mg/kg minimized COX-2 SN and SC. Carveol at 10 mL/kg declined COX-2 SN and SC. At 20 mL/kg, it subsided COX-2 in the SN and SC, as shown in Figure 8A. Berbamine at 1 mg/kg decreased TNF-α in the SN and SC. At 5 mg/kg, it downregulated TNF-α in the SN and SC, and at 15 mg/kg, it diminished TNF-α in the SN and SC. Bergapten at 25 mg/kg decreased TNF-α in the SN and SC. At 50 mg/kg, it downregulated TNF-α in the SN and SC. Bergapten at 100 mg/kg dropped TNF-α in the SN and SC. Carveol at 10 mL/kg decreased the expression of TNF-α in the sciatic nerve. At 20 mL/kg it downregulated TNF-α in the SN and SC as shown in Figure 8B. Berbamine at 1 mg/kg decreased p-NF-κb in the SN and SC. At 5 mg/kg it downregulated NF-κb in the SN and SC, and at 15 mg/kg it reduced NF-κbin the SN and SC. Bergapten at 25 mg/kg decreased p-NF-κb in the sciatic nerve. At 50 mg/kg it downregulated NF-κb in the SN and with *p* < 0.05 in the SC. Bergapten at 100 mg/kg decreased the expression of NF-κb in the SN and SC. Carveol at 10 mL/kg decreased NF-κb in the SN and SC. At 20 mg/kg it downregulated NF-κb in the SN and SC as shown in Figure 8C.

### 3.9. Western Blot Findings

**Figure 9:**
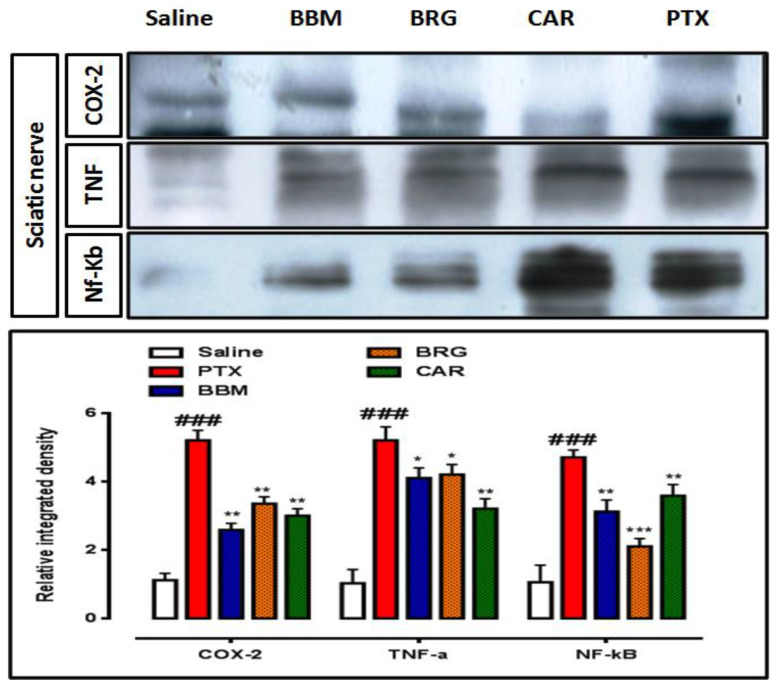

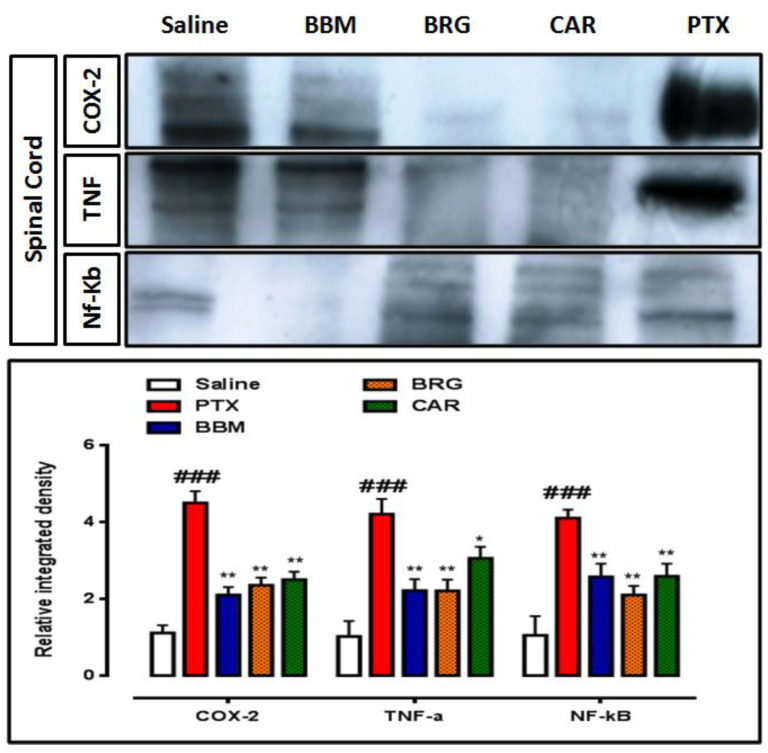
Representation of the effects of berbamine, bargepten, and carveol on the expression of COX-2, TNF-α, and NF-κb, quantified by using a Western blot sciatic nerve and spinal cord. The data are expressed as the mean ± SEM, *n* = 6. One-way ANOVA with posthoc Tukey’s test. * *p* < 0.05, ** *p* < 0.01, and *** *p* < 0.001 indicate a significant difference vs. PTX, and ^###^ *p* < 0.001 indicate a significant difference vs. saline. Morphological data were analyzed by ImageJ software. Bar 50 µm, magnification 40×.

**Figure 10:**
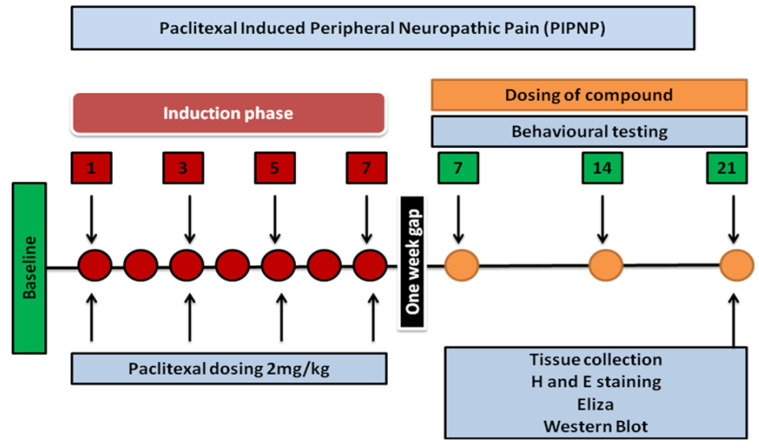
The scheme of the study. The baseline reading was collected on day0. The induction phase was started with administration of 2 mg/kg of PTX on day 1 and was continued on alternative days until day 7. After one week, the behavioral studies were conducted to confirm the development of neuropathy. At the same time, treatment was started and was continued until day 21.

The inflammatory markers were subsequently studied using Western blot analysis in both the sciatic nerve and the spinal cord, and the findings are shown in Figure 9 and Figure 10. All inflammatory indicators were considerably elevated in the collected samples compared to saline, according to the findings. COX-2, TNF-, and NF-b were all inhibited by berbamine. COX-2, TNF-, and p-NF-b were all inhibited by bergapten. In comparison to the PTX group, carveol reduced COX-2, TNF-, and NF-b in the sciatic nerve sample. Berbamine inhibited COX-2, TNF-, and NF-b. Bergapten attenuated COX-2, TNF-α, and NF-κb. Carveol decreased the expression of COX-2, TNF-α, and NF-κb in the spinal cord sample vs. PTX.

## 4. Discussion

One of the most severe and disabling adverse medication reactions in more than 75% of cancer regimens is paclitaxel-induced peripheral neuropathy (PIPN) [32]. There are several ways to cause paclitaxel-induced peripheral neuropathy in rats, including a preventive technique (neuropathy is produced while the drug is present) and a pre-existing neuropathy method (neuropathy is established in the absence of drug). The potential of three natural compounds (BBM, BAR, and CAR) in chemotherapy-induced discomfort was secured using the pre-existing neuropathy method. To cause neuropathy, paclitaxel was administered at a dosage of 2 mg/kg on days 1, 3, and 5. Treatment was not started until neuropathy had developed and been verified by performing behavioral tests.

The pain threshold was supported by the following factors: body weight, mechanical hypersensitivity, latency before falling, and thermal pain feeling. As the number of cancer patients rises, there is an urgent need to create medications with a lower toxicity and higher efficacy profile in order to improve patient quality of life and reverse PIPN. Berberine [33], icariin [34], and melatonin [35] are only a few of the medications that have been studied for this purpose. Many natural products are renowned for their wide range of therapeutic potential, and researchers have worked hard to develop such substances as important nutrients to treat a variety of illnesses [36,37]. Moreover, as pain is the protective mechanism alarming us about the proper measure to be taken for the initiated problem, so people utilize different approaches to relieving pain associated with chronic illness [38]

In order to discover compounds for effective reversal of PIPN, we selected three natural compounds, BBM, BRG, and CAR. They were chosen to see how they may affect PIPN caused by PTX. The selection of these compounds was based on the fact that they are anti-inflammatory and neuroprotective [14,15,17] in nature and can be investigated in paclitaxel-associated chronic inflammation.

Several markers were investigated, including nuclear factor-2 and poly ADP-ribose polymerase, both of which are implicated in anticancer-induced neuropathic pain [39]. NF-kB, nitric oxide, COX-2, IL-1, IL-6, and TNF-α have also been shown to be overexpressed in PIPN [40]. Toll-like receptors (TLR-1, TLR-2, and TLR-4) and the transient receptor potential family of ion channels (TRPV1 and TRPV4) have been discovered, demonstrating their active role in chemotherapy-induced peripheral neuropathy [18,41,42]. It is generally established that oxidative stress is linked to all neuropathies. The induction of oxidative stress by paclitaxel is a well-known mechanism for the formation and maintenance of PIPN [42]. Its use causes the depletion of GSH and GST, which are implicated in blocking hazardous chemical metabolites in chemotherapy-induced pain [43,44,45]. In addition to GSH and GST, other harmful variables such as LPO and iNOS [42] are shown to be raised following PTX treatment and have a role in enhancing pain feeling. We tested the effects of three natural substances on paclitaxel-induced peripheral neuropathy. Figure 10 depicts the theme of the current research work’s strategy.

The sciatic nerve and spinal cord were collected for further analysis. The disruption in latency of falling body weight and mechanical hypersensitivity are all key components of paclitaxel-induced neuropathy [46,47,48]. The behavioral study that we conducted on our drugs showed that berbamine, bergapten, and carveol were able to repair the behavioral deficit that was caused by paclitaxel-induced peripheral neuropathic pain. These findings are presented in Figure 1, Figure 2, Figure 3 and Figure 4. The findings were determined to be in line with those of previous research. Numerous studies have concentrated their attention on the neuronal level of the sciatic nerve and spinal cord in paclitaxel-induced neuropathy and have found morphological abnormalities at both levels [49]. In addition, we investigated the morphology of two different tissues and came to the conclusion that PTX played a role in the progression of the disease by mediating neuronal degeneration, disrupting the development of myelin sheaths, and causing vacuolation. The fact that treatment with the natural substances mentioned above restored the damage caused by PTX suggests that these substances could be categorized as neuroprotective agents. This is demonstrated in Figure 5, Figure 6 and Figure 7. Molecular research is primarily responsible for determining the expression of a wide variety of different types of inflammatory mediators and transcription factors. These molecular methods include the enzyme linked immunosorbent assay as well as the Western blot, both of which are utilized to evaluate the levels of endogenous marker expression [50,51,52]. The upregulation of inflammatory markers such as COX-2, TNF-a, and NF-kB, which are typically linked to chemotherapy-induced neuropathic pain, were also investigated as part of our research. Experiments using ELISA and Western blot demonstrated that PTX increased the expression of all of the pathogenic indicators that were previously mentioned. In an animal model of paclitaxel-induced neuropathic pain, treatment with the aforementioned treatments led to a reduction in the expression of COX-2, TNF-a, and NF-kb, which in turn led to a reduction in the severity of the pain.

## 5. Conclusions

This research explored that berbamine, bergapten, and carveol reversed paclitaxel-induced neuropathic pain by correcting behavioral deficits and increasing protective markers like GSH and GST, which are the key components involved in scavenging free radicals, thus protecting the body from their harmful consequences. The natural compounds decreased damaging factors like LPO and iNOS and downregulated overexpressed inflammatory mediators like COX-2, TNF-, and NF-kB, as evidenced by ELISA and Western blotting. This potential of the natural compounds (berbamine, bergapten, and carveol) makes them promising agents for neuroprotective effects. To broaden the list of medications useful in the therapy of neuropathic pain, further research is needed to clarify the pharmacokinetics and pharmacodynamics profile, as well as stability and dose form.

## Figures and Tables

**Table 1:** Effects of berbamine (BBM), bargepten (BRG), and carveol (CAR) on the expression of GSH, GST, iNOS, and LPO in the sciatic nerve. The data expressed as the mean ± SEM, *n* = 6. One-way ANOVA with posthoc Tukey’stest.

Group	GSH (µmol/mg of Protein)	GST (µmol CDNB Conjugate/min/mg of Protein)	iNOS (µmol/mg of Protein)	LPO (nmol/TBARS/mg of Protein)
Saline (10 mL/kg)	48.22 ± 2.1	43.88 ± 1.5	34.22 ± 3.1	62.43 ± 1.8
PTX (2 mg/kg)	7.22 ± 1.7 ^###^	10.53 ± 2.6 ^###^	105.32 ± 3.2 ^###^	286.66 ± 2.2 ^###^
PTX+BBM (15 mg/kg)	30.29 ± 2.2 **	36.10 ± 3.4 **	66.21 ± 1.6 *	112.36 ± 2.8 *
PTX+BRG (100 mg/kg)	24.24 ± 2.2 **	29.14 ± 2.4 **	78.11 ± 2.2 *	128.10 ± 1.8 *
PTX+CAR (20 mL/kg)	28.14 ± 1.2 **	33.50 ± 1.4 **	56.11 ± 2.6 *	132.16 ± 3.8 *

* p < 0.05 and ** *p* < 0.01 indicate a significant difference vs. PTX, and ^###^ *p* < 0.001 indicates a significant difference vs. saline.

**Table 2:** Effects of berbamine (BBM), bargepten (BRG), and carveol (CAR) on the expression of GSH, GST, iNOS, and LPO in the spinal cord. The data are expressed as the mean ± SEM, *n* = 6. One-wayANOVA with posthoc Tukey’s test.

Group	GSH (µmol/mg of Protein)	GST (µmol CDNB Conjugate/min/mg of Protein)	iNOS (µmol/mg of Protein)	LPO (nmol/TBARS/mg of Protein)
Saline (10 mL/kg)	43.22 ± 1.8	35.71 ± 2.1	41.35 ± 1.2	62.33 ± 1.3
PTX (2 mg/kg)	9.41 ± 1.5 ^###^	7.53 ± 3.4 ^###^	98.32 ± 2.3 ^###^	195.68 ± 1.6 ^###^
PTX+BBM (15 mg/kg)	33.21 ± 1.4 **	26.14 ± 3.2 **	52.41 ± 2.5 **	76.66 ± 1.0 **
PTX+BRG (100 mg/kg)	25.21 ± 2.6 **	26.12 ± 3.2 **	73.41 ± 1.5 **	113.22 ± 1.8 ***
PTX+CAR (20 mL/kg)	29.14 ± 0.6 **	22.22 ± 1.2 **	63.11 ± 1.5 **	103.36 ± 2.0 ***

** *p* < 0.01 and *** *p* < 0.001 indicate a significant difference vs. PTX, and ^###^ *p* < 0.001 indicates a significant difference vs. saline.

## Data Availability

All the data are incorporated in the manuscript.

## Abbreviations

Berbamine	(BBM)
Bergapten	(BRG)
Carveol	(CAR)
Paclitaxel	(PTX)
Glutathione	(GSH)
Glutathione S-transferase	(GST)
Inducible nitric oxide synthase	(iNOS)
Lipid peroxidase	(LPO)
Hematoxylin and eosin	(H and E)
Immunohistochemistry	(IHC)
Cyclooxygenase	(COX-2)
Tumor necrosis factor-alpha	(TNF-α)
Nuclear factor kappa B	(NF-κb)
Enzyme-linked immunosorbant assay	(Elisa)
Phosphate buffer saline	(PBS)
Hydrogen peroxide	(H_2_O_2_)
5,5′-dithio-bis-2-nitrobenzoic acid	(DTNB)
2,4-Dinitrochlorobenzene	(CDNB)
Diaminobenzidine peroxidase	(DAB)
Trichloroacetic acid	(TCA)
Research and ethical committee	(REC)
Intraperitoneally	(i.p)
Sciatic nerve	(SN)
Spinal cord	(SC)
Nitric oxide	(NO)
Horseradish peroxidase	(HRP)
Heat latency	(HL)
Falling	(LF)
Paw withdrawal threshold	(PWT)
Paclitaxel induced peripheral neuropathic pain	(PIPNP)
Chemotherapy-induced peripheral neuropathic pain	(CIPNP)
Toll-like receptors	(TLR)
Transient receptor potential	(TRP)

## References

[1] Postma T., Vermorken J., Liefting A., Pinedo H., Heimans J. (1995). Paclitaxel-induced neuropathy. Ann. Oncol..

[2] Zajączkowska R., Kocot-Kępska M., Leppert W., Wrzosek A., Mika J., Wordliczek J. (2019). Mechanisms of chemotherapy-induced peripheral neuropathy. Int. J. Mol. Sci..

[3] Augusto C., Pietro M., Cinzia M., Sergio C., Sara C., Luca G., Scaioli V. (2008). Peripheral neuropathy due to paclitaxel: Study of the temporal relationships between the therapeutic schedule and the clinical quantitative score (QST) and comparison with neurophysiological findings. J. Neurooncol..

[4] Rowinsky E.K., Donehower R.C. (1995). Paclitaxel (taxol). N. Engl. J. Med..

[5] Xue X., Liu H., Wang S., Hu Y., Huang B., Li M., Su J. (2022). Neutrophil-erythrocyte hybrid membrane-coated hollow copper sulfide nanoparticles for targeted and photothermal/ anti-inflammatory therapy of osteoarthritis. Compos. Part B Eng..

[6] Boyette-Davis J.A., Walters E.T., Dougherty P.M. (2015). Mechanisms involved in the development of chemotherapy-induced neuropathy. Pain Manag..

[7] Klein I., Lehmann H.C. (2021). Pathomechanisms of paclitaxel-induced peripheral neuropathy. Toxics.

[8] Zhang H., Li Y., de Carvalho-Barbosa M., Kavelaars A., Heijnen C.J., Albrecht P.J., Dougherty P.M. (2016). Dorsal root ganglion infiltration by macrophages contributes to paclitaxel chemotherapy-induced peripheral neuropathy. J. Pain.

[9] Caillaud M., Thompson D., Toma W., White A., Mann J., Roberts J.L., Damaj M.I. (2022). Formulated Curcumin Prevents Paclitaxel-Induced Peripheral Neuropathy through Reduction in Neuroinflammation by Modulation of α7 Nicotinic Acetylcholine Receptors. Pharmaceutics.

[10] Li X., Yang S., Wang L., Liu P., Zhao S., Li H., Jiang Y., Guo Y., Wang X. (2019). Resveratrol inhibits paclitaxel-induced neuropathic pain by the activation of PI3K/Akt and SIRT1/PGC1α pathway. J. Pain Res..

[11] Kaur S., Muthuraman A. (2019). Ameliorative effect of gallic acid in paclitaxel-induced neuropathic pain in mice. Toxicol. Rep..

[12] Zhang X.-L., Cao X.-Y., Lai R.-C., Xie M.-X., Zeng W.-A. (2019). Puerarin relieves paclitaxel-induced neuropathic pain: The role of Nav1. 8 β1 subunit of sensory neurons. Front. Pharmacol..

[13] Memariani Z., Abbas S.Q., ul Hassan S.S., Ahmadi A., Chabra A. (2021). Naringin and naringenin as anticancer agents and adjuvants in cancer combination therapy: Efficacy and molecular mechanisms of action, a comprehensive narrative review. Pharmacol. Res..

[14] Zhao Y., Lv J., Chen J., Jin X., Wang M., Su Z., Wang L., Zhang H. (2016). Berbamine inhibited the growth of prostate cancer cells in vivo and in vitro via triggering intrinsic pathway of apoptosis. Prostate Cancer Prostatic Dis..

[15] Raquet N., Schrenk D. (2009). Relative photomutagenicity of furocoumarins and limettin in the hypoxanthine phosphoribosyl transferase assay in V79 cells. Chem. Res. Toxicol..

[16] Srivastava J., Lambert J., Vietmeyer N. (1996). Medicinal Plants: An Expanding Role in Development.

[17] Alvi A.M., Al Kury L.T., Alattar A., Ullah I., Muhammad A.J., Alshaman R., Li S. (2021). Carveol attenuates seizure severity and neuroinflammation in pentylenetetrazole-kindled epileptic rats by regulating the Nrf2 signaling pathway. Oxidative Med. Cell. Longev..

[18] Li Y., Zhang T., Wang Z., Liang H., Wu Z., Li J., Ou-Yang J., Zhu B. (2022). Transcranial Focused Ultrasound Stimulation of Periaqueductal Gray for Analgesia. IEEE Trans. Biomed. Eng..

[19] Bardin L., Malfetes N., Newman-Tancredi A., Depoortere R. (2009). Chronic restraint stress induces mechanical and cold allodynia, and enhances inflammatory pain in rat: Relevance to human stress-associated painful pathologies. Behav. Brain Res..

[20] Huang C., Hu Z.-P., Long H., Shi Y.-S., Han J.-S., Wan Y. (2004). Attenuation of mechanical but not thermal hyperalgesia by electroacupuncture with the involvement of opioids in rat model of chronic inflammatory pain. Brain Res. Bull..

[21] Ba X., Wang J., Zhou S., Luo X., Peng Y., Yang S., Hao Y., Jin G. (2018). Cinobufacini protects against paclitaxel-induced peripheral neuropathic pain and suppresses TRPV1 up-regulation and spinal astrocyte activation in rats. Biomed. Pharmacother..

[22] Joshi R.P., Negi G., Kumar A., Pawar Y.B., Munjal B., Bansal A.K., Sharma S.S. (2013). SNEDDS curcumin formulation leads to enhanced protection from pain and functional deficits associated with diabetic neuropathy: An insight into its mechanism for neuroprotection. Nanomed. Nanotechnol. Biol. Med..

[23] Imran M., Al Kury L.T., Nadeem H., Shah F.A., Abbas M., Naz S., Khan A.-U., Li S. (2020). Benzimidazole containing acetamide derivatives attenuate neuroinflammation and oxidative stress in ethanol-induced neurodegeneration. Biomolecules.

[24] Guedes R.P., Dal Bosco L., da Rosa Araújo A.S., Belló-Klein A., Ribeiro M.F.M., Partata W.A. (2009). Sciatic nerve transection increases gluthatione antioxidant system activity and neuronal nitric oxide synthase expression in the spinal cord. Brain Res. Bull..

[25] Iqbal S., Shah F.A., Naeem K., Nadeem H., Sarwar S., Ashraf Z., Imran M., Khan T., Anwar T., Li S. (2020). Succinamide derivatives ameliorate neuroinflammation and oxidative stress in scopolamine-induced neurodegeneration. Biomolecules.

[26] Kumar K.S., Hsieh H.W., Wang S.-Y. (2010). Anti-inflammatory effect of lucidone in mice via inhibition of NF-κB/MAP kinase pathway. Int. Immunopharmacol..

[27] Mohsin Alvi A., Tariq Al Kury L., Umar Ijaz M., Ali Shah F., Tariq Khan M., Sadiq Sheikh A., Nadeem H., Khan A.-U., Zeb A., Li S. (2020). Post-treatment of synthetic polyphenolic 1,3,4 oxadiazole compound A3, attenuated ischemic stroke-induced neuroinflammation and neurodegeneration. Biomolecules.

[28] Ali A., Shah F.A., Zeb A., Malik I., Alvi A.M., Alkury L.T., Rashid S., Hussain I., Ullah N., Khan A.U. (2020). NF-κB inhibitors attenuate MCAO induced neurodegeneration and oxidative stress—A reprofiling approach. Front. Mol. Neurosci..

[29] Hassan S.S.U., Muhammad I., Abbas S.Q., Hassan M., Majid M., Jin H.Z., Bungau S. (2021). Stress driven discovery of natural products from actinobacteria with anti-oxidant and cytotoxic activities including docking and admet properties. Int. J. Mol. Sci..

[30] Al Kury L.T., Zeb A., Abidin Z.U., Irshad N., Malik I., Alvi A.M., Khalil A.A.K., Ahmad S., Faheem M., Khan A.-U. (2019). Neuroprotective effects of melatonin and celecoxib against ethanol-induced neurodegeneration: A computational and pharmacological approach. Drug Des. Devel. Ther..

[31] Majid M., Farhan A., Asad M.I., Khan M.R., Hassan S.S.U., Haq I.U., Bungau S. (2022). An Extensive Pharmacological Evaluation of New Anti-Cancer Triterpenoid (Nummularic Acid) from Ipomoea batatas through In Vitro, In Silico, and In Vivo Studies. Molecules.

[32] Sisignano M., Baron R., Scholich K., Geisslinger G. (2014). Mechanism-based treatment for chemotherapy-induced peripheral neuropathic pain. Nat. Rev. Neurol..

[33] Singh J., Saha L., Singh N., Kumari P., Bhatia A., Chakrabarti A. (2019). Study of nuclear factor-2 erythroid related factor-2 activator, berberine, in paclitaxel induced peripheral neuropathy pain model in rats. J. Pharm. Pharmacol..

[34] Gui Y., Zhang J., Chen L., Duan S., Tang J., Xu W., Li A. (2018). Icariin, a flavonoid with anti-cancer effects, alleviated paclitaxel-induced neuropathic pain in a SIRT1-dependent manner. Mol. Pain.

[35] Ambriz-Tututi M., Rocha-González H.I., Cruz S.L., Granados-Soto V. (2009). Melatonin: A hormone that modulates pain. Life Sci..

[36] Bano I., Horky P., Abbas S.Q., Majid M., Bilal A.H.M., Ali F., Bungau S. (2022). Ferroptosis: A New Road towards Cancer Management. Molecules.

[37] Xie Y.G., Zhao X.C., ul Hassan S.S., Zhen X.Y., Muhammad I., Yan S.K., Jin H.Z. (2019). One new sesquiterpene and one new iridoid derivative from Valeriana amurensis. Phytochem. Lett..

[38] Laktasic Zerjavic N., Hrkic E., Zagar I., Delimar V., Kovac Durmis K., Spoljarić Carevic S., Peric P. (2021). Local cryotherapy, comparison of cold air and ice massage on pain and handgrip strength in patients with rheumatoid arthritis. Psychiatr. Danub..

[39] Komirishetty P., Areti A., Yerra V.G., Ruby P., Sharma S.S., Gogoi R., Sistla R., Kumar A. (2016). PARP inhibition attenuates neuroinflammation and oxidative stress in chronic constriction injury induced peripheral neuropathy. Life Sci..

[40] Yerra V.G., Negi G., Sharma S.S., Kumar A. (2013). Potential therapeutic effects of the simultaneous targeting of the Nrf2 and NF-κB pathways in diabetic neuropathy. Redox Biol..

[41] Pascual M., Baliño P., Aragón C.M., Guerri C. (2015). Cytokines and chemokines as biomarkers of ethanol-induced neuroinflammation and anxiety-related behavior: Role of TLR4 and TLR2. Neuropharmacology.

[42] Hara T., Chiba T., Abe K., Makabe A., Ikeno S., Kawakami K., Utsunomiya I., Hama T., Taguchi K. (2013). Effect of paclitaxel on transient receptor potential vanilloid 1 in rat dorsal root ganglion. PAIN®.

[43] Ishii N., Tsubouchi H., Miura A., Yanagi S., Ueno H., Shiomi K., Nakazato M. (2018). Ghrelin alleviates paclitaxel-induced peripheral neuropathy by reducing oxidative stress and enhancing mitochondrial anti-oxidant functions in mice. Eur. J. Pharmacol..

[44] Mir O., Alexandre J., Tran A., Durand J.-P., Pons G., Treluyer J.-M., Goldwasser F. (2009). Relationship between GSTP1 Ile105Val polymorphism and docetaxel-induced peripheral neuropathy: Clinical evidence of a role of oxidative stress in taxane toxicity. Ann. Oncol..

[45] Mccormick B., Lowes D., Colvin L., Torsney C., Galley H. (2016). MitoVitE, a mitochondria-targeted antioxidant, limits paclitaxel-induced oxidative stress and mitochondrial damage in vitro, and paclitaxel-induced mechanical hypersensitivity in a rat pain model. BJA Br. J. Anaesth..

[46] Naveed M., Ullah R., Khan A., Shal B., Khan A.U., Khan S.Z., Khan S. (2021). Anti-neuropathic pain activity of a cationic palladium (II) dithiocarbamate by suppressing the inflammatory mediators in paclitaxel-induced neuropathic pain model. Mol. Biol. Rep..

[47] Pawar S.H., Upaganlawar A.B., Upasani C.D. (2021). Attenuation of Hyperalgesia and Allodynia by some Phenolic Acids in Paclitaxel Induced Neuropathy. bioRxiv.

[48] Sullivan K.A., Grant C.V., Jordan K.R., Vickery S.S., Pyter L.M. (2021). Voluntary wheel running ameliorates select paclitaxel chemotherapy-induced sickness behaviors and associated melanocortin signaling. Behav. Brain Res..

[49] Son D.B., Choi W., Kim M., Go E.J., Jeong D., Park C.-K., Kim Y.H., Lee H., Suh J.-W. (2021). Decursin alleviates mechanical allodynia in a paclitaxel-induced neuropathic pain mouse model. Cells.

[50] Yardım A., Kandemir F.M., Çomaklı S., Özdemir S., Caglayan C., Kucukler S., Çelik H. (2021). Protective effects of curcumin against paclitaxel-induced spinal cord and sciatic nerve injuries in rats. Neurochem. Res..

[51] Kashem M.A., Li H., Toledo N.P., Omange R.W., Liang B., Liu L.R., Li L., Yang X., Yuan X.-Y., Kindrachuk J. (2019). Toll-like interleukin 1 receptor regulator is an important modulator of inflammation responsive genes. Front. Immunol..

[52] Mahmood F., Khan J.A., Mahnashi M.H., Jan M.S., Javed M.A., Rashid U., Bungau S. (2022). Anti-Inflammatory, Analgesic and Antioxidant Potential of New (2 S, 3 S)-2-(4-isopropylbenzyl)-2-methyl-4-nitro-3-phenylbutanals and Their Corresponding Carboxylic Acids through In Vitro, In Silico and In Vivo Studies. Molecules.

